# Robust High-dimensional Bioinformatics Data Streams Mining by ODR-ioVFDT

**DOI:** 10.1038/srep43167

**Published:** 2017-02-23

**Authors:** Dantong Wang, Simon Fong, Raymond K. Wong, Sabah Mohammed, Jinan Fiaidhi, Kelvin K. L. Wong

**Affiliations:** 1 Department of Computer and Information Science, Univeristy of Macau, SAR, Macau; 2 School of Computer Science and Engineering, University of New South Wales, Australia; 3 Department of Computer Science, Lakehead University, Thunder Bay, Canada; 4 School of Medicine, University of Western Sydney, New South Wales, Australia

## Abstract

Outlier detection in bioinformatics data streaming mining has received significant attention by research communities in recent years. The problems of how to distinguish noise from an exception and deciding whether to discard it or to devise an extra decision path for accommodating it are causing dilemma. In this paper, we propose a novel algorithm called ODR with incrementally Optimized Very Fast Decision Tree (ODR-ioVFDT) for taking care of outliers in the progress of continuous data learning. By using an adaptive interquartile-range based identification method, a tolerance threshold is set. It is then used to judge if a data of exceptional value should be included for training or otherwise. This is different from the traditional outlier detection/removal approaches which are two separate steps in processing through the data. The proposed algorithm is tested using datasets of five bioinformatics scenarios and comparing the performance of our model and other ones without ODR. The results show that ODR-ioVFDT has better performance in classification accuracy, kappa statistics, and time consumption. The ODR-ioVFDT applied onto bioinformatics streaming data processing for detecting and quantifying the information of life phenomena, states, characters, variables and components of the organism can help to diagnose and treat disease more effectively.

Due to the popularity of Internet-of-things, smart cities, sensing applications, big data and cloud computing, data collection has become more prevalent than before. A large amount of data is being gathered in time-series, which arrives at the collector channeling to the analyzer or decision making component at high speed in large volume. Along the data journey, the data are prone to be perturbed with noises which may appear occasionally through multiplexing, synchronization and different media/equipment of different transmission/operation qualities. One of the objectives of data pre-processing in data mining process is to pick out the outliers and possibly cleanse the training data before loading them into the model construction. It is known that raw data is likely to contain noise, which adversely affects the speed, accuracy and robustness of data analysis.

Outlier detection has received much concern in the traditional data mining field. Relatively, this topic is less being looked into in bioinformatics data stream mining. The operational environment of data stream mining is in fact more susceptible harsh outdoor operational conditions. Hence abnormalities in data are likely to occur as results of typographical errors or measurement errors. By definition, outliers are data, which have values that are either too large or too small being exceptionally different from the average. It is like a double-edged sword in the sense that outliers can be useful in applications that are designed to identify the abnormal such as frauds, or rare events in prediction models; accurate prediction of outliers can potentially prevent devastating consequence. On the other hand, outliers can cause serious performance degradation in supervised learning, should outliers be used as a part of the training data. Put simply, it is a dilemma on how outliers should be handled: to discard or to keep. This is assumed the operational environment such as high speed bioinformatics data stream mining, the context is completely free from human expert intervention. From the pure computational perspective, how these outliers should be treated in the pre-processing, is a tricky research question.

The difficulties of detecting outlier from the big continuous dataset lie in whether noise is perceived as good or bad element in training up a classification model. A simplified but core principle is considered here: if an outlier is detected to occur once or twice, it is likely to be noise. The singular occurrence does not warrant embracing it into the classification model, as it is not worth to devise an internal decision path in the model for just a singular instance. It should be just discarded. If the outlier appears twice or more in instances, there might be some significance about them; therefore, some attention should be paid to them. In this case, further observation is needed to decide whether these outliers contribute or disrupt the learning patterns for the classification model. One easy measure is the consistency of the outlier patterns, which are formed by multiple occurrences of outliners.

The objective of this paper is to improve the learning algorithm in bioinformatics data stream mining especially the preceding part for handling noise data using statistical measure for detection and contradiction measure for possible removal. There are also other approaches to detect and remove noise from dataset, such as preventing overfit by using a validation set during classifier training, pruning insignificant leaves from the decision tree, or identifying and removing misclassified instances by k-nearest neighbors. In our method, we opt for mechanisms that are simple, light-weight and operate first. Ideally, we choose to sort out the outliers before they enter into the model-training phrase.

## Discussion

Very fast decision tree is one of the incremental decision tree methods that can reduce the training time for large time series dataset. The incremental optimized VFDT (ioVFDT) is using the Hoeffding Tree in node-splitting control^1^. It makes use of Hoeffding bounds or addistive Chernoff bounds^2^ 
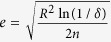
 to provide an approximate model with statistically guaranteed error bounds, which *n* is the independent instances with range *R*, with confidence 1−*δ*, the true mean of *r* is at least 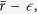 and 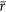 is the observed mean of the instances. The bound determines the node splitting with high probability but using smallest number of *N* examples from big data. The tree path from root to a leaf is computed by the regression pattern. That means whenever a tree node need to split a new leaf, the calculation should start from the root to the end of new leaf. The more data to process, the more the split of new node may have, and the greater the amount of computation consumption. The method to load and process stream dataset is by sliding window. With an arriving sample each time, the time complexity will be *O*(1) for every time. Suppose that *O(w*) is to be the complexity for ioVFDT in learning the continuous dataset, where *w* is the window size. It is horizontal to faults when selecting a wrong window size. It may represent a very accurate result for current state if the window length is too narrow. But the data analysis accuracy may influence by noisy data while too wide windows result in the effects of outlier. Nevertheless, learning mechanism with unload full time series data and using the Hoeffding bounds to determines the node splitting for only use smallest number of *N* samples is likely missing detect the outlier point from the whole streaming dataset.

Although there are many ways to deal with outlier by preprocessing and removing the noisy data, such as continuous monitoring of distance-based outlier (MCOD), which an object χ is an outlier if there are less than κ objective lying at distance at most Ρ from χ^3^. The synthetic minority over sampling technique (SMOTE), which fuzzy rough prototype selection algorithm can remove noisy instances from the imbalanced dataset^4^. The isolate forest is another unsupervised learning outlier algorithm that explicit isolate anomalies instances instead of profiles normal points^5^. But all of those approaches are not combined with learning process. That means we should go through the outlier procedure to clean out all the data, then use the clean data to do machine learning. One deficiency of this technique is that we are unable to handle the continuous dataset because times series data is coming out by time, and the traditional method is to finish outlier processing before performing machine learning. Another one is that it is time consuming. The total running time for data mining is the sum of time deplete of outlier and machine learning.

The location based outlier detection methods such as mean, mode and median usually unstable and difficult for calculating. Mean based approaches can be greatly affected by extreme values and sometimes the mean value calculated among the whole dataset may not be an actual ‘meaningful’ value. Mode based approaches are not affected by extreme values and can be obtained for qualitative data. But some set of data may have more than one models or other sets even do not have model values. The requirement of rank order dataset for median based methods may not suit for processing time series bioinformatics dataset. The way we choice is dispersion based outlier detection measure. Compared with variance and standard deviation based outlier detection approaches, interquartile range do not have the complex calculation and much easier to compute. It is more stable and not affect by the extreme values among the continuous dataset since the influence of the extremes of a distribution are eliminated. Although the advantages of using the interquartile range rather than other approaches for the measurement of the spread of a dataset is that interquartile range is not sensitive to outliers, but it can be disadvantage in some case as well, such as clustering analytics. For this point, we mainly focus on processing the data exception in bioinformatics dataset classification, such as EEG, EMG, and diabetes data, etc.

One effective solution to achieve the lowest computation is by combining outlier removal technique and machine learning together. This means making a quick data preprocess to detect and remove the outlier from noisy dataset, and meanwhile passing the clean data to machine learning. This method can fundamentally reduce the time consumption, split of tree nodes and the affection of window length selection, which we have mentioned before.

## Proposed Methods

### Outlier Detection

**Figure 1:**
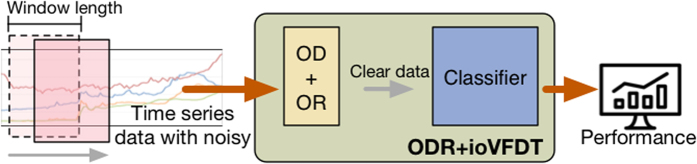
ODR-A-ioVFDT Model.

The time series dataset was loaded by sliding window. Let *R* = {*i*_1_, *i*_2_, *i*_3_, …, *i*_*n*_} be a set of continuous raw data which may contain noise. *ω* is the window length that loads *W*_1_ = {*i*_1_, *i*_2_, *i*_3_, …, *i*_*ω*_}, *W*_1_ ∈ *R* a bunch of data from a round of data load is *W*_2_ = {*i*_1+*m*_, *i*_2+*m*_, *i*_3+*m*_, …, *i*_*ω*+*m*_}, *W*_2_ ∈ *R* (Fig. 1). So the *Z* = {*W*_1_, *W*_2_, *W*_3_, …, *W*_*n*_}, where *Z* ⊆ *R*. Outlier detection and removal approach (ODR-A) based on adaptive interquartile range (IQR) which is computed from cumulative data so far, since data stream is unbounded. Suppose *Q*_1_ is the lower quartile and *Q*_3_ is upper quartile of one window size data *W*_*n*_.

We calculate the interquartile range by integrating the probability density function (PDF):


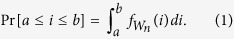


Here, *W*_*n*_ has density *f*_*Wn*_, where *f*_*Wn*_ is a non-negative Lebesgue-integrable function. As for the data is time series that load by sliding window. So the *f*_*Wn*_ is continuous at *W*_*n*_, *F*_*Wn*_ is the cumulative distribution function (CDF) of *W*_*n*_:


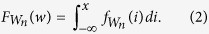


We can think of *f*_*Wn*_(*i)di* as being the probability of *W*_*n*_ falling within the infinitesimal interval [*i, i* + *di*]. So the lower extreme value is lower than the integral of the PDF (function 1) from −∞ to *Q*_1_ equals 0.25 and upper extreme value is higher than the integral from −∞ to *Q*_3_ equals 0.75. Then, *Q*_1_ and *Q*_3_ can be defined as follow:


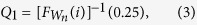



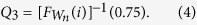


The outlier that is detected within the sliding window is *o*_*n*_, where *o*_*n*_ ∈ *W*_*n*_. Here, *O* = {*o*_1_, *o*_2_, *o*_3_, …, *o*_*n*_} is the set of outlier detect from the whole dataset, and *O* ⊆ R. As the diversified huge volume dataset *R* has been processed by stream mining, the initial outlier detection disposed result *O* will be given a secondary treatment for contradiction estimation. The threshold *β* is the maximum contradiction detects value of outlier comparison, it determines the final value of Local Outlier factor (LOF). The upper threshold is the percentage of total data that the data value higher than *Q*_3_ + (*β* × IQR) and lower is the threshold less than *Q*_1_ + (*β* × IQR). If the data size of *O* < *LOF*_*min*_, *O* will be removed from original dataset and collected into misclassified database. If the size of *O* > *LOF*_*min*_, *O* will be regarded as a group of special cases but still belong to original dataset.

**Figure 2:**
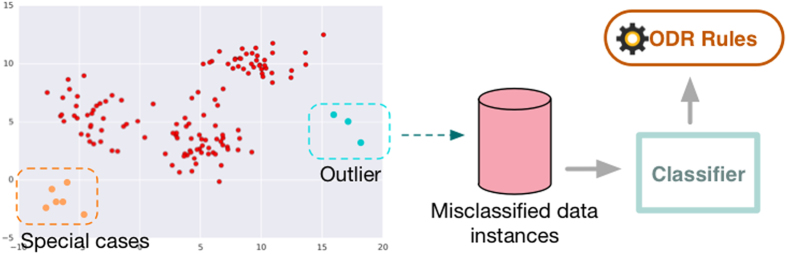
ODR Processing Model.

The clear dataset without outlier is *C* = {*c*_1_, *c*_2_, *c*_3_, …, *c*_*n*_}, *C* ⊆ R but *O* ⊄ *C*. The clear data *C* will be passed to ioVFDT classifier to study once ODR-A finished filtering one sliding window raw data (Fig. 1). The *O* will be collected into misclassified database (Fig. 2) for the classifier (ioVFDT) to learn and generate the outlier detection and removal rules (ODR-R).

**Figure 3:**
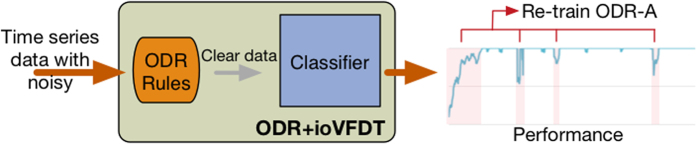
ODR-R-ioVFDT Model.

**Figure 4:**
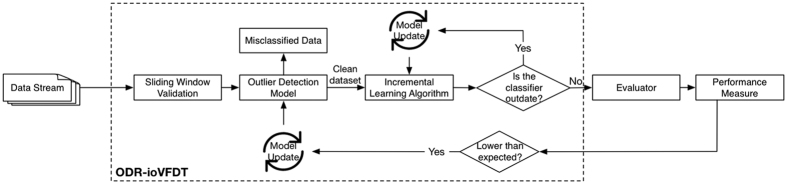
ODR-ioVFDT Model. The time series data was loaded by the sliding window. Outlier will be picked out by the ODR model, and then collected into misclassified database. Clean data will be passed through ioVFDT classifier for decision tree building. The prediction error ***Err***_***n***_ will be calculate for evaluate the classifer efficiency. The performance where lower than the expectation will send feedback to outlier and classifier model, the model update will be needed.

The essential factor for using ODR-A or ODR-R is determined by the accuracy of learning performance. The restrict accuracy value is *Acc*_*min*_ if the current accuracy (*Acc*_*cur*_) is lower than *Acc*_*min*_ the ODR-A will be executed to collect outlier instances and update ODR-R. Otherwise the outlier will implement ODR-R for data processing (Figs 3 and 4). The total time consume for preprocessing raw data is *T*_*ODR*_ where *T*_*ODR*_ = *t*_*ODR−A*_ + *t*_*ODR−R*_.


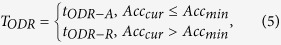


### ioVFDT Algorithm

To achieve fast learning characteristics, Hoeffding’s inequality *e* provides an upper bound on the probability that only partial examples will be needed to achieve certain level of confidence. Incremental optimization very fast decision tree (ioVFDT) is based on Hoeffding Tree (HT) that using the Hoeffding Bound (HB) in node-splitting control to reduce the unbound tree size constructing. Using Hoeffding’s Lemma and Markov’s inequality can proof the Hoeffding bounds or addistive Chernoff bounds:


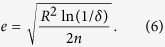


The cost function **Θ**(*HT*_*R*_) for the tree building solution is shown as (7):


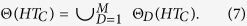


The ioVFDT node splitting mechanism is a kind of heuristic learning method by using greedy search approach to train the continuous coming dataset *C*_*i*_. Suppose there are *δ* attributes in the clean dataset *C*. The node splitting determined by greedy search calculation of the whole attributes *δ*_*i*_ values. 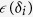 is the heuristic function that takes a node *n* and returns a non-negative real number that is an estimates of the path cost from node *n* to a goal node. 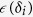 have to be calculated for each attribute. The tree path from root to a leaf is computed by the regression pattern. When tree node splitting for a new underneath leaf, the decision tree’s structure is changing, and tree depth may become deeper. If we use the heuristic estimation function to inspect the splitting node state 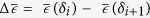, then the tree size function is shown in (8):


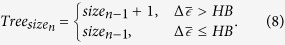


As we have mentioned previously, *C* is the clear dataset without outlier. Suppose Ω is the practical solution for *C*, which can implement a certain optimization objective. And Ω ⊆ 2^*c*^. *M* is the number of substances in the optimization problem. The previous work given that when *M* = 3, it costs slightest to build compact tree model that poises the tree size, predictive precision and time consume. When continuous clean dataset *C* = {*c*_1_, *c*_2_, *c*_3_, …, *c*_*n*_} arrives, the predictive accuracy *Acc*_*n*_ is dynamically shifting with sample size *n* rising in an incremental learning process:


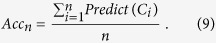


The *Acc*_*n*_ is always compared with *Acc*_*min*_. If *Acc*_*n*_ is lower in value than *Acc*_*min*_, ODR-A model will be run and this modifies the ODR-R model until *Acc*_*n*_ higher than *Acc*_*min*_. The prediction error is *Err*_*n*_ = 1 − *Acc*_*n*_. The restrict maximum error value is *Err*_*max*_ = 1 − *Predict(C*)_*min*_. If the current error value *Err*_*cur*_ is higher than *Err*_*max*_. The previous period *o*_*n*_ will be released to iOVFD for re-learning. If the re-learning result is worse than current’s, which *Err*_*re*_ ≥ *Err*_*cur*_. Then, the *o*_*n*_ will be considered as noise. Otherwise, if *Err*_*re*_ < *Err*_*cur*_, *o*_*n*_ will be referred to as the worthwhile cases, which may be the orphan disease that shows relatively abnormal data record compares to the normal measure values.

### ODR-ioVFDT Algorithm

The outlier detection and removal approach combined with ioVFDT algorithm is presented in the pseudo-codes here. The input parameters are given in Table 1. The procedure of ODR is given in Table 2. The ioVFTD building is given in Table 3, which presents the reliable ioVFDT learning process.

## Experiments and Datasets

The experimental platform is a Java package of ODR-ioVFDT, which is integrated with the Massive Online Analysis (MOA) toolkit that is a free Java benchmarking platform for data stream experiments. The running environment for the experimentation is a series of Unix-based graphical interface operating system (Mac OS X) with 2.9 GHz Intel core i7 CPU and 8 GB ROM. We used the default values of parameters for algorithms as recommended by MOA and the data loading window size is kept at 1000 in all experiments.

The five different scenarios of continuous dataset are those that possess diverse features, they were tested with a two decision-tree-based bioinformatics data stream mining algorithms and three classical classification algorithms, namely ODR-ioVFDT, ioVFDT, KNN, NB and SVM. The kernel-based SVM is a non-probabilistic binary linear classifier. It has a regularization parameter to avoid over-fitting and an approximation to a bound on the test error rate^6^,^7^. But the kernel models can be quite sensitive to over-fitting the model selection criterion^8^,^9^. A non-parametric and instance-based learning method, KNN, is one of the simplest classification and regression algorithms in machine learning. It has the high robust and good effective in processing noisy and large data^10^. But KNN needs to set the parameter K value, the computation cost also quite high. The KNN approach is difficult to estimate which type of distance and attribute to use can perform the best result^11^. Naïve Bayes is a simple, and quicker converges than discriminative probabilistic classifier with strong independence assumptions between the features. But it a very strong assumption on coming data distribution^12^,^13^, and cannot learn interactions between features neither^14^. The incremental optimized very fast decision tree takes the mean of Hoeffding bound values for tie-breaking. It builds up the tree use the smallest number of samples and does not require a full dataset to be stored in memory.

### Data Description

The five datasets reflect the typical uses of data mining on biomedical applications. They range from gene processing, biosignal streams classification to diabetes insulin treatment control. The records to those datasets are measured and collected in different varieties, such as the 3Vs in the context of big data - volume, variety and velocity. The diversified datasets for five different scenarios (Table 4) will process different features in the experiment with ODR-ioVFDT and other bioinformatics data stream mining approaches. In order to validate the outlier detection ability without sliding window restriction, we picked the dataset samples with lengths ranging from hundreds to millions. And those datasets also contain varied attributes and classes. All the datasets are obtained from real-world recourses those are available downloaded from UCI Machine Learning Repository^15^, Knowledge Extraction based on Evolutionary learning open source platform^16^. The five datasets are briefly described as follow:

### sEMG for Basic Hand Movements Data Set (EM)

The EMG data were collected at a sampling rate of 15 Hz and 500 Hz with a Butterworth Band Pass filter for Basic Hand movements, included 2 databases of surface electromyography signals of 6 hand movements using Delsys’ EMG System. The subjects were asked to perform six daily hand grasps movements repeatedly, which are spherical, tip, palmar, lateral, and cylindrical. The dataset total has 1,800 instance, and 3,000 attributes.

### Lymphoblastic leukemia Data Set (LL)

The lung cancer for gene dataset has been divided into six diagnostic groups (BCR-ABL, E2A-PBX1, Hyperdiploid >50, MLL, T-ALL and TEL-AML1), and one that contains diagnostic samples that did not fit into any one of the above groups (labeled as Others). There are 12,558 genes and 1,962 instances.

### Thyroid Disease Databases (TD)

This dataset was collected from the Garavan Institute. The task is to detect is a given patient is normal (1) or suffers from hyperthyroidism (2) or hypothyroidism (3). There are 7,200 instance and 21 attributes. All the data value are numeric. With the complex and enormous continuous data, it can simulate the process of real-time big data generation and processing.

### EEG Eye State Data Set (EE)

The continuous EEG measurement with the Emotiv EEG Neuroheadset dataset was collected by Baden-Wuerttemberg Cooperative State University, Stuttgart, Germany. The eye state was detected via a camera during the EEG measurement and added later manually to the file after analyzing the video frames. It has 14,980 instance and 15 attributes. There are two classification of the dataset that ‘1’ indicates the eye-closed state and ‘0’ is the eye-open state. This dataset is suitable for evaluating our outlier approach because it contains a few missing values, the instances are continuous numeric with both small and larger values.

### Diabetes Data Set (DD)

Diabetes dataset pertains to a large sample size, and contains varied format of attributes value, which has around one million set for each 70 patient that is covering several weeks’ to months’ worth of outpatient care. The datasets contain ten attributes, which are ‘date/time’, ‘time from the last NPH insulin’, ‘last NPH dose’, ‘time from last regular insulin’, ‘last regular dose’, ‘type of bgm’, ‘bgm value’, ‘GMT’ and ‘Hypo’. The results can reflect the robust of different process methodology. Since the dataset has a large number of attributes and instances, it can help to test the outlier affection for the decision tree building.

## Results

The five data stream mining algorithms were tested in five different scenarios continuous dataset. According to the design concept of ODR-ioVFDT, when stream data gets loaded into the ODR-ioVFDT, the ODR will detect outliers over the data stream and move outliers to the misclassified database. The outlier factor was selected from 1 to 5. So Table 5 shows the number of outliers that have been collected into the misclassified database. The smaller threshold *β* set, the wider range ODR will detect from the dataset. When we process ODR for each tested classifier, it can be easily found that the ODR help improve the classification accuracy compared with the performances without ODR. The major performance results are Classification accuracy, Kappa statistics, Time elapsed, Tree size.

### Classification accuracy

**Figure 5:**
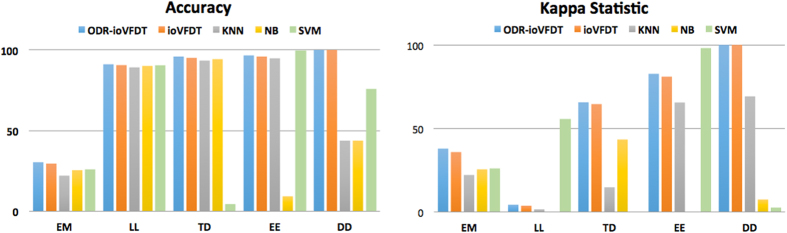
Classification Accuracy & Kappa Statistics for Five Algorithms.

**Figure 6:**
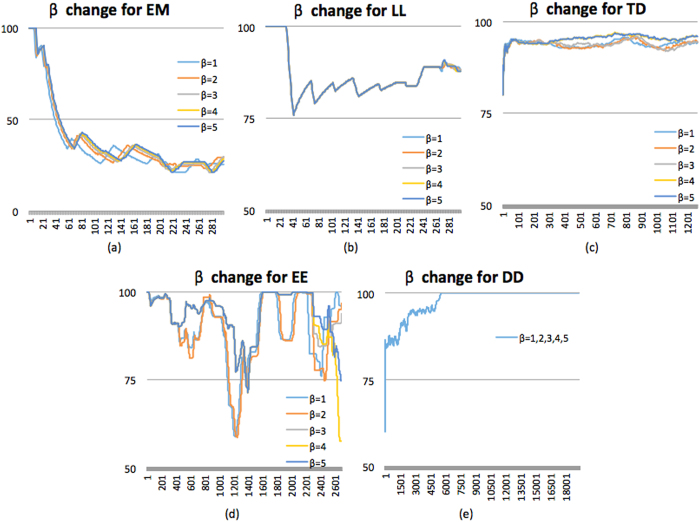
The ODR-ioVFDT classification accuracy with outlier threshold *β* changed in five dataset.

As ODR-ioVFDT is the optimized algorithm based on ioVFDT, we will compare the classification accuracy with ODR-ioVFDT and ioVFDT firstly. From Fig. 5 we can find that ODR-ioVFDT shows a better performance on data stream classification than ioVFDT, in all the five datasets. The higher accuracy preponderance of ODR-ioVFDT is not obvious in datasets LL and EE, although both algorithms performed nearly perfect at 91.10% for ODR-ioVFDT in LL and 96.60% for ioVFDT in EE. The accuracy performance in dataset LL and DD illustrates that the data processing of ODR-ioVFDT is similar to ioVFDT in some datasets, in which they have the same accuracy trend and approximate value. SVM shows good results on dataset LL and EE in 90.60% and 99.70% classification accuracy respectively. But we can find that SVM cannot apply universally since its performance becomes unstable in dataset TD with 4.50% accuracy. And NB has the same defect that performance in dataset EE. From Table 6, we observed that the synchronized ODR preprocessing and classifier combination for stream dataset learning shows better results in all cases of datasets. Different values of *β* and how they influence the accuracy as resulted by using ODR-ioVFDT over different dataset are shown in Fig. 6. Whatever the selected value of threshold *β* is, the learning and classification accuracy has significantly improved. It shows (in Table 6) more persuasive results in dataset DD since NB and SVM learning accuracy increased nearly 40 percentages.

### Kappa statistics

Here, Fig. 5 and Table 7 shows the kappa statistics for all algorithms on each dataset. Kappa statistics specifies how generalized a prediction model is. It is a measure of the inter-ratter agreement for qualitative of the prediction of the algorithm performs, and is generally thought to be a more robust measure rather than a simple percent agreement calculation. This means that the kappa statistics value will be higher, and the training model will be more reliable. It is clear to us that the ODR-ioVFDT and ioVFDT demonstrate outstanding performance in dataset DD with perfect scores of 100% in both accuracy and kappa statistics. Although their kappa statistics are relatively higher than the other algorithms in dataset EM and DT, the values are still off the mark. NB performances in dataset LL and EE with 0, and it even achieved a high value in precision, whereas the accuracy values are not credible. In EE, SVM obtained the highest kappa values of 98.21%. In contrast, ODR-ioVFDT is robust in terms of classification, which can be applied to a variety of situations.

### Time elapsed

**Figure 7:**
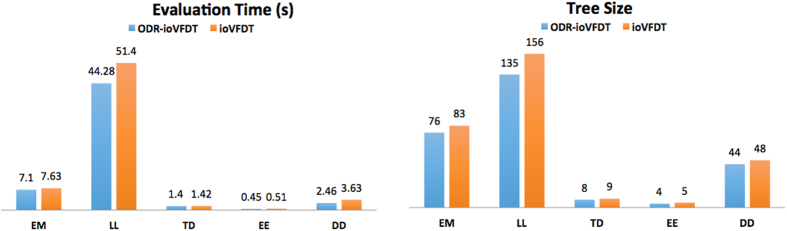
Time Consume and tree size comparison between ODR-ioVFDT & ioVFDT.

The compromise of ODR-ioVFDT is that it is time consuming due to the overhead of outlier detection and handling. From Table 8 and Fig. 7 we can observe that ODR-ioVFDT performs faster in processing in all continuous datasets than ioVFDT does. Although NB and SVM shows good results in time consume, it is noticed that NB and SVM have failure classification and low kappa statistic in some datasets. Note that KNN cost significant processing time in all kinds of data stream processing.

### Tree size

The tree sizes are tabulated in Table 9 and charted in Fig. 7. The size of a decision tree is the number of nodes in the tree. If a decision tree is fully-grown, it may lose some generalization capability, which is known as overfitting since one of the reason overfitting happens is that the presence of outliers. According to our hypothesis, the outlier detection and removal preprocess will help to reduce extra tree node generation, thereby keeping the decision trees compact. From the experiment results on all the datasets, the hypothesis can be confirmed true given that ODR-ioVFDT results in a smaller tree size when compared to that of the ioVFDT.

### Related Work

There are many ways to categorize outlier detection approaches. To illustrate by the class objective, one-class classification outlier detection approach proposed by Tax^17^. The artificial outlier is generated by normal instances that are trained by a one-class classifier. Then the combination of one-class and support vector data description algorithms is given to achieve a boundary decision between normal and outlier samples. But the drawback of one-class classification is not able to handle multi-objective dataset. Thus the genetic programming for one-class classifier proposed by Loveard and Cielsieski^18^ aims to apply for diverse formalisms in its evolutionary processing. Since the multifarious dataset with diversity classes take over most type of dataset, the outlier detection approach for multi-objective is in wilderness demand^19^. The instances that pertain to the misclassified (ISMs) filtering method exhibit a high level of class overlap for similar instance implemented by Michael R. Smith *et al*.^20^. The approach is based on two measure heuristics, one is k-Disagreeing Neighbors (kDN) for taking space of local overlap instances, and another is Disjunct Size (DS) for dividing instances by covering instance of largest disjunct among the dataset. Although this method performs well on outlier reduction, but high cost of time is the biggest drawback of ISMs.

The pattern learning outlier detection models are usually categorized into clustering, distance-based, density-based, probabilistic and information-theoretic. Wu, Shu, and Shengrui Wang^21^ are using an information-theoretic model to share an interesting relationship with other models. A concept of holoentropy that takes both entropy and total correlation into consideration to be the outlier factor of an object, which is solely determined by the object itself and can be updated efficiently. This method constrain the maximum deviation allowed from them normal model. It will be reported as an outlier if it has the large difference. Zeng, Xiangxiang, Xuan Zhang, and Quan Zou^22^ gave a biological interaction networks for finding out the information between gene, protein, miRNA and disease phtnotype and predicting potential disease-related miRNA based on networks. Ando, Shin^23^ is giving a scalable minimization algorithm base on information bottleneck formalization that exploits the localized form of the cost function over individual clusters. Bay, Stephen D and Mark Schwabacher^24^ are displaying a distance-based model that uses a simple nested loop algorithm. It will give near linear time performance in the worst case. Knorr, Edwin M^25^ gives a K-nearest neighbor distribution of a data point to determine whether it is an outlier. Xuan, Ping *et al*.^26^ gave a prediction method HDMP based on weighted K most simialer neighbors to find the similarity between disease and phenotype^27^. Yousri, Noha A.^28^ displayed an approach that is clustering considering a complementary problem to outlier analysis. A universal set of clusters is proposed which combines clusters obtained from clustering, and a virtual cluster for the outlier. It optimized clustering model to purposely detect outliers. Breunig, Markus M *et al*.^29^ used density-based model to define its outlier score, in which local outlier factor degree depends on how isolated the object is related to the surrounding neighborhood. Cao, Feng *et al*.^30^ also present a density-based micro-cluster to summarize the clusters with arbitrary shape, which guarantees the precision of the weights of the micro-clusters with limited memory. Yuen, Ka-Veng, and He-Qing Mu^31^ gave a probabilistic method for robust parametric identification and outlier detection in linear regression approach. Brink, Wikus *et al*.^32^ derived a Gaussian noise model for outlier removing. The probabilistic approach is almost analogous to those clustering algorithms whereby the process of fitting values are used to quantify the outlier scores of data points.

Our outlier identify method of incremental optimized very fast decision tree with outlier detection and removal (ODR-ioVFDT) is an nonparametric optimized decision tree classifier based on probability density that is excusive of the outlier detection and removal first, and in the meantime, send the clean data flow to ioVFDT^33^,^34^. This algorithm aims to reduce the running time of classification and increase the accuracy of prediction by making a quick time-series preprocessing of dataset.

## Conclusion

In this paper, we design and implement ODR-ioVFDT model, which combines outlier detection and remove all contradicting outlier instances from the incremental optimized very fast decision tree. A prediction/classification model should embrace outliers for general recognition powers – in case outliers may show up again though it is not often, so the model is able to recognize it, instead of removing (or neglecting) it in model induction during supervised learning. In the experiments, we used five different scenarios of real-world datasets to test five classification algorithms, and ODR-ioVFDT shows better performances in terms of classification accuracy, kappa statistics, and is more time consuming than the others. The preprocess of the continuous noisy data for bioinformatics data stream mining meets our hypothesis of reducing the time consumption to as minimal as possible, split of tree nodes and the affection of window length selection. The contribution of this paper is improving the learning algorithm in bioinformatics data stream mining, especially the preceding part for handling noise data by using statistical measure, detection and contradiction measure for possible removal. The ODR-ioVFDT algorithm applied onto bioinformatics streaming data processing for detecting and quantifying the information of life phenomena, states, characters, variables and components of the organism can help to improve diagnosis and make more effective treatment for disease.

We demonstrate the feasible potential of the proposed ODR-ioVFDT in identifying data outliers from bioinformatics data stream, with enhanced overall data mining performance. Our generic model, as reported in this paper provides the fundamentals of the design, which can be extended to develop specialized applications for disease prevention, diagnosis and treatment. For future work, we can incorporate metaheuristic algorithms such as particle swarm optimization for automatically selecting the optimal configuration values for both ODR thresholds and ioVFDT parameters. Although the new method may increase the time complexity for processing data stream, its computational overhead can possibly be justified by further improvements in decision rules building and classification accuracy. More validation works and fine-tuning are needed in experimenting new models and testing the hypothesis in the future, which can be based on our fundamental ODR-ioVFDT framework.

## Additional Information

**How to cite this article:** Wang, D. *et al*. Robust High-dimensional Bioinformatics Data Streams Mining by ODR-ioVFDT. *Sci. Rep.* **7**, 43167; doi: 10.1038/srep43167 (2017).

**Publisher's note:** Springer Nature remains neutral with regard to jurisdictional claims in published maps and institutional affiliations.

## Figures and Tables

**Table 1:** Input & Output Parameters.

Input:	
*R*:	A set of raw data stream
*δ*:	The number of attributes that dataset contains
*ω*:	Sliding window length
*m*:	The window moving length
*Acc*_*min*_:	The restriction of accuracy value
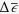 :	The heuristic estimation function
*Ω*:	The practical solution for tree building
Output:	
*O*:	Outlier dataset detect from dataset R
*HT*:	A decision tree
*Acc*_*n*_:	The accuracy of tree learning process

**Table 2:** The Procedure of ODR.

1:	The continuous dataset *R* arrives
2:	**IF** *W*_*n*_ ! = EOF
3:	**FOR** each window size data *W*_*n*_
4:	Compute *Q*_*1*_ = [*F*_*Wn*_(*i*)]^−1^(0.25)
	*Q*_*3*_ = [*F*_*Wn*_(*i*)]^−1^(0.75)
5:	Detect outlier *Q*_*1*_ + (*β* * IQR) > *o*_*n*_ && *o*_*n*_ > *Q*_3_ + (*β* * IQR)
6:	//based on function (3) & (4)
7:	Collect *o*_*n*_ to set *O*
8:	Remove *O* from *R* get clean dataset *C*
9:	Call function ioVFDT(*C*)
10:	//Sent *C* to ioVDFT
11:	*Err*_*ckur*_ = *iOVFDT(C*)
12:	*Err*_*max*_ = *Err*[*max*]
13:	Call function iOVFDT(*o*_*n*_)
14:	*Err*_*re*_ = *iOVFDT(o*_*n*_)
15:	**IF** *Err*_*re*_ ≥ *Err*_*cur*_
16:	*o*_*n*_ ∈ *OSS*
17:	End **IF**
18:	End **FOR**
19:	End **IF**

**Table 3:** ioVFDT Building.

1:	Let iOVFDT be a tree with a single leaf (root)
2:	**FOR** all training and testing examples *C* **DO**
3:	Sort example into leaf using iOVFDT
4:	**IF** *δ*_*i*_ *mod* *δ*_*min*_ = 0
5:	Compute 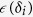 for each attribute
6:	Let *δ*_*i*_ be attribute with highest 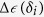
7:	Let *δ*_*i+*1_ be attribute with highest 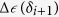
8:	Compute Hoeffding bounds 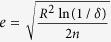
9:	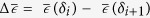
10:	If *δ*_*i*_ ≠ *δ*_*i*+1_ and 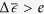
11:	**FOR** all branches of the split **DO**
12:	Add a new leaf with initialized sufficient statistics
13:	End **FOR**
14:	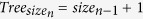
15:	End **IF**
16:	End **IF**
17:	Compute 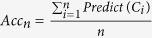
18:	*Err*_*n*_=1−*Acc*_*n*_
19:	Return *Err*_*n*_
20:	End **FOR**

**Table 4:** Dataset Description.

Name	Abbreviation	Sample size	No. of attributes	No. of classes
sEMG for Basic Hand Movements	EM	1,800	3,000	6
Lymphoblastic leukemia	LL	1,962	12,559	6
Thyroid Disease Databases	TD	7,200	21	3
EEG Eye State	EE	14,980	15	2
Diabetes data set	DD	100,000	9	2

**Table 5:** Outlier Collected by Misclassified Database.

	*β* = 1	*β* = 2	*β* = 3	*β* = 4	*β* = 5
EM	303	114	58	33	16
LL	294	210	138	84	48
TD	850	279	129	53	10
EE	999	499	272	170	55
DD	3030	3030	3030	3030	3030

**Table 6:** ODR Preprocessing for Five Dataset by Five Algorithms.

(a)				
EM	KNN	NB	SVM	ioVFDT
*β* = 0	22.10	25.50	25.90	29.55
*β* = 1	20.20	25.30	27.30	**30.39**
*β* = 2	21.80	**26.90**	**27.50**	29.83
*β* = 3	**22.70**	26.20	26.20	29.21
*β* = 4	22.50	26.00	26.10	29.37
***β***** = 5**	**22.20**	**25.70**	**25.90**	**29.48**
**(b)**				
**LL**	**KNN**	**NB**	**SVM**	**ioVFDT**
*β* = 0	89.20	90.10	90.60	90.60
*β* = 1	89.70	89.80	90.10	88.20
*β* = 2	**90.10**	**90.60**	90.60	**91.10**
*β* = 3	90.10	90.40	**91.00**	90.10
*β* = 4	89.90	90.20	90.60	90.40
*β* = 5	90.00	90.40	90.80	91.10
**(c)**				
**TD**	**KNN**	**NB**	**SVM**	**ioVFDT**
*β* = 0	93.40	94.30	4.40	95.10
*β* = 1	**93.50**	**94.40**	4.40	94.30
*β* = 2	93.00	94.10	**4.50**	94.10
*β* = 3	93.20	94.10	4.50	94.00
*β* = 4	93.10	94.10	4.50	**95.90**
*β* = 5	93.40	94.30	4.40	95.90
**(d)**				
**EE**	**KNN**	**NB**	**SVM**	**ioVFDT**
*β* = 0	94.8	9.30	99.70	95.90
*β* = 1	94.80	9.70	99.70	93.60
*β* = 2	94.80	**9.80**	**99.70**	93.90
*β* = 3	**94.80**	9.80	99.70	92
*β* = 4	94.80	9.70	99.70	**96.6**
*β* = 5	94.80	9.50	99.70	96.6
**(e)**				
**DD**	**KNN**	**NB**	**SVM**	**ioVFDT**
*β* = 0	92.80	43.80	75.80	100
*β* = 1, 2, 3, 4, 5	93.20	86.60	75.40	100.00

**Table 7:** Kappa statistics.

	EM	LL	TD	EE	DD
ODR-ioVFDT	37.9	4.3	65.65	82.7	100
ioVFDT	35.81	3.7	64.57	80.98	100
KNN	22.1	1.55	14.72	65.51	69.19
NB	25.5	0	43.33	0	7.44
SVM	25.9	55.83	0	98.21	2.39
Reference	Remarks				
0.0~20.0%	Slight				
21.0~40.0%	Fair				
41.0~60.0%	Moderate				
61.0~80.0%	Substantial				
81.0~100.0%	Almost perfect				

**Table 8:** Time Elapsed (s).

	EM	LL	TD	EE	DD
ODR-ioVFDT	7.1	44.28	1.4	0.45	2.46
ioVFDT	7.63	51.4	1.42	0.51	3.63
KNN	294.02	1672.48	3.13	7.55	19.63
NB	4.69	20.1	0.11	0.17	1.95
SVM	2.34	7.53	0.06	0.11	1.73

**Table 9:** Tree Size for Five Datasets.

	EM	LL	TD	EE	DD
ODR-ioVFDT	76	135	8	4	48
ioVFDT	83	156	9	5	44
